# Integrated analysis of mRNA and miRNA profiles revealed the role of miR-193 and miR-210 as potential regulatory biomarkers in different molecular subtypes of breast cancer

**DOI:** 10.1186/s12885-020-07731-2

**Published:** 2021-01-18

**Authors:** Adriane F. Evangelista, Renato J. Oliveira, Viviane A. O. Silva, Rene A. D. C. Vieira, Rui M. Reis, Marcia M. C. Marques

**Affiliations:** 1 Molecular Oncology Research Center, Barretos Cancer Hospital, Barretos, São Paulo, 14784-400 Brazil; 2 Department of Mastology and Breast Reconstruction, Barretos Cancer Hospital, Barretos, São Paulo, 14784-400 Brazil; 3 Life and Health Sciences Research Institute (ICVS), Health Sciences School, University of Minho, Braga, 4710-057 Portugal; 4 ICVS/3B’s-PT Government Associate Laboratory, Braga/Guimarães, 4710-057 Portugal; 5 Tumor Biobank, Barretos Cancer Hospital, Barretos, São Paulo, 14784-400 Brazil; 6 Barretos School of Health Sciences, FACISB, Barretos, São Paulo, 14784-400 Brazil

**Keywords:** Breast cancer, miR-193, miR-210, MiRNA-mRNA interaction, Cell proliferation, Cell migration

## Abstract

**Background:**

Breast cancer is the most frequently diagnosed malignancy among women. However, the role of microRNA (miRNA) expression in breast cancer progression is not fully understood. In this study we examined predictive interactions between differentially expressed miRNAs and mRNAs in breast cancer cell lines representative of the common molecular subtypes. Integrative bioinformatics analysis identified miR-193 and miR-210 as potential regulatory biomarkers of mRNA in breast cancer. Several recent studies have investigated these miRNAs in a broad range of tumors, but the mechanism of their involvement in cancer progression has not previously been investigated.

**Methods:**

The miRNA-mRNA interactions in breast cancer cell lines were identified by parallel expression analysis and miRNA target prediction programs. The expression profiles of mRNA and miRNAs from luminal (MCF-7, MCF-7/AZ and T47D), HER2 (BT20 and SK-BR3) and triple negative subtypes (Hs578T e MDA-MB-231) could be clearly separated by unsupervised analysis using HB4A cell line as a control. Breast cancer miRNA data from TCGA patients were grouped according to molecular subtypes and then used to validate these findings. Expression of miR-193 and miR-210 was investigated by miRNA transient silencing assays using the MCF7, BT20 and MDA-MB-231 cell lines. Functional studies included, xCELLigence system, ApoTox-Glo triplex assay, flow cytometry and transwell inserts were performed to determine cell proliferation, cytotoxicity, apoptosis, migration and invasion, respectively.

**Results:**

The most evident effects were associated with cell proliferation after miR-210 silencing in triple negative subtype cell line MDA-MB-231. Using in silico prediction algorithms, *TNFRSF10* was identified as one of the potential regulated downstream targets for both miRNAs. The *TNFRSF10C* and *TNFRSF10D* mRNA expression inversely correlated with the expression levels of miR-193 and miR210 in breast cell lines and breast cancer patients, respectively. Other potential regulated genes whose expression also inversely correlated with both miRNAs were *CCND1*, a known mediator on invasion and metastasis, and the tumor suppressor gene *RUNX3*.

**Conclusions:**

In summary, our findings identify miR-193 and miR-210 as potential regulatory miRNA in different molecular subtypes of breast cancer and suggest that miR-210 may have a specific role in MDA-MB-231 proliferation. Our results highlight important new downstream regulated targets that may serve as promising therapeutic pathways for aggressive breast cancers

**Supplementary Information:**

The online version contains supplementary material available at (10.1186/s12885-020-07731-2).

## Background

Breast cancer (BC) is the most commonly occurring malignancy in women worldwide with more than 2 million new cases diagnosed in 2018 [1]. BC is characterized by high levels of intra and inter-tumor heterogeneity that impact several levels, including variation in histological features, together with differences in response to treatment and to patient survival outcomes [2]. An understanding of inter-patient differences is crucial for relating breast cancer biology to new targeted therapeutics. Variations in the expression of established prognostic and predictive biomarkers togther with hormone receptor status remains a challenge for clinical management and use of targeted therapies [3].

Previous studies based on global gene expression analyses have provided additional insights into the complexities of BC therapeutics. Perou et al. [4, 5] initially classified breast tumors into four molecular subtypes based on their gene expression profiles. The known intrinsic molecular subtypes of breast cancer were extensively characterized, and showed significant differences in their incidence, relative risk factors, prognosis and treatment sensitivity [6, 7]. The most recent guidelines have supported a classification based on five molecular subtypes: luminal A, luminal B, luminal B HER2 positive, HER2-enriched and triple negative. Additionally, RNA-based multigene expression assays have been developed to define other molecular subtypes showing some evidence of clinical utility [8, 9]. However, there are distinct limitations with classification schemes solely based on gene expression. Moreover, the reproducibility of these methods has been questioned [10, 11], drawing attention to the necessity for the identification of new types of biomarkers that can more rigorously distinguish between the various molecular subtypes of BC.

Recent findings have drawn attention to the role microRNAs (miRNAs) may play as novel biomarkers and their future potential as therapeutic targets in cancer [12–14]. MiRNAs are particularly promising due to their molecular stability, their ease of detection by non-invasive methods and their ability to provide improved subtype classification [12–14]. MiRNAs are a class of small non-coding regulatory RNAs that are involved in controlling gene expression at the posttranscriptional level [15, 16]. These regulatory transcripts are short, single-stranded RNA sequences (approximately 19–23 nucleotides) that are able to modulate gene expression and mediate a variety of physiological processes. They have direct involvement in several diseases and are known to play an important role in cancer [17, 18]. In BC, several miRNAs have been reported to be involved in prognosis, metastasis and response to therapy [19, 20]. However, details of the molecular mechanisms underlying the regulation of miRNA expression in breast cancer are not fully understood.

In this study, we investigated the global expression profiles of miRNA from breast cancer cell lines and TCGA datasets derived from different molecular subtypes of BC to identify miRNAs candidates associated with specific molecular subtypes. In parallel, we performed an integrative analysis of mRNA profiles to identify any putative miRNA targets for a deeper understanding the regulatory impact of miRNAs on the cancer biology of BC. The expression of downstream genes affected by regulatory miRNAs may influence important key molecular pathways that could serve as targets for cancer therapy. We selected genes involved in general transcription and regulation processes that could also affect apoptosis and cell proliferation as both pathways hold therapeutic promise in BC. Finally, we further investigated the functional roles of the selected miRNAs for a better understanding of their role in BC. Further studies are needed to gain more insights into miR-193 and miR-210 targets and their associated signaling pathways.

## Methods

### Cell culture and RNA isolation

Seven human BC cell lines from different molecular subtypes and one breast normal cell were utilized, as follows: luminal (MCF-7, MCF-7/AZ and T47D), HER2 (BT20 and SK-BR3), triple negative (Hs578T e MDA-MB-231) and normal control cell (HB4A). All the cells were obtained from the American Type Culture Collection (ATCC; Manassas, VA, USA) and were maintained in Dulbecco’s modified Eagle’s medium (DMEM) supplemented with 10% fetal bovine serum (FBS) and 1% penicillin/streptomycin solution (Gibco, Invitrogen), at 37^∘^C and 5% CO2 atmosphere. Cell lines authentication was performed using in-house kit for Short tandem repeat (STR) fragments profiling by the Molecular Diagnostics at the Barretos Cancer Hospital as previously reported [21]. Mycoplasma detection and control is determined every 15 days of culture. Total RNA was extracted by Trizol™(Invitrogen) according to the manufacturer’ instructions, with an additional overnight precipitation step at −20^∘^C with isopropanol (Merck). RNA quantification and purity were carried out in a Qubit quantitation platform (Invitrogen) and RNA integrity was assessed by microfluidic electrophoresis using a 2100 Bioanalyzer with RNA 6000 nanochips (Agilent Technologies, Santa Clara, CA, USA). Samples presenting 260/280 and 260/230 ratios of 1.8-2.0 and RNA integrity number (RIN) ≥9.0 were used.

### mRNA and microRNA microarrays

Hybridizations of mRNAs and miRNAs were performed as previously reported by our group [22–24]. The oligo microarrays technology used for gene expression assays was the SurePrint G3 Human Gene Expression v3 8x60K oligo microarrays (G4851C, Agilent Technologies) and the Human miRNA Microarray Kit (V3) (8x15K-G4471A, Agilent Technologies) was used to assess the expression of miRNAs. For both microarrays, the total RNA amount (200 ng) and the one color (cyanine-3, Cy3) Quick Amp labeling kit (Agilent) was used. The hybridization steps varied according the manufacturer’ instructions, with 17 hours at 65^∘^C for mRNA and 24 hours at 55^∘^C for miRNA microarrays. Images were acquired using an Agilent DNA microarray scanner with SureScan technology (Agilent Technologies).

### Microarray data analysis

The raw microarray expression data were obtained using the Feature Extraction software v.12.0 (Agilent Technologies) and submitted to the R environment to be analyzed using dedicated packages from Bioconductor [25, 26]. Median signals were used as intensity values in both microarrays. Normalization was performed using the quantile method with the limma package [27]. To identify the miRNAs differentially expressed between the BC cell lines and control cells we performed a rank products analysis considering both *P*-value and pfp (positive false predictions) ≤0.05 and also ANOVA (p ≤0.01 with Bonferroni correction) for multiple conditions [28, 29]. Analysis of potential mRNA-miRNA interactions was performed using bidirectional analysis of mirDIP, i.e. the lists of both differentially expressed molecules were considered in the analysis [30]. The target genes were independently selected by all the algorithms provided by this platform using the selection criteria of occurrence common to at least four algorithms. We only considered the top 1% of target genes, including those identified by the Cancer Gene Index data (NCI) as candidates for being involved in breast cancer. To further determine how the selected genes could be associated with breast cancer and the molecular pathways related to these genes, we used the plugin ReactomeFI on Cytoscape version 3.6.0 [31, 32].

### TCGA patient’s selection and validation

Validation of potential miRNA expression, mRNA-miRNAs interactions and targets that were predicted by our microarray data analysis of BC cell lines was carried out using TCGA databases level 3 [33]. The TCGA data repository had samples containing miRNA sequencing, derived from 1198 patient tumors. According to the TCGA guidelines, the datasets used present no limitations or restrictions at the moment. All clinical and associated molecular data were retrieved using RTCGA and FirebrowseR packages [34, 35]. The patient tumors were stratified according to their molecular subtypes (luminal, HER2 or triple negative) using the information available regarding the molecular status of ER, PR and HER2 and Ki-67 markers. Only concordant markers between HER2 immunohistochemistry and FISH were used. Patients with missing biomarker data for at least one miRNA were also excluded. Patients could be classified for luminal (n = 279), HER2 (n = 54) and triple-negative (n = 123). Data from the histologically normal breast tissue adjacent to the tumor (NT; n = 57) of the same patients were used as normal control group.

### Transient transfection of microRNA inhibitors and quantiative real-Time PCR

The hsa-mir-210 and hsa-miR-193a-3p miRCURY LNA™ microRNA inhibitors (Exiqon) were transfected into BT20/MDA-MB-231 and BT20/MCF-7 cells, respectively, using INTERFERin (Polyplus Transfection), according to the manufacturer’s protocol. Cells without treatment with inhibitors (NC group) and transfected with miRCURY LNA™ microRNA antisense Control A (Exiqon) (Scramble group) were used as negative controls of the transfection. The miRNA expression before and after transfection procedures were assessed by RT-qPCR. Reverse transcription to the sequence of miR-193a-3p and miR-210 was performed with total RNA (10 ng) using TaqMan® Small RNA Assays (Thermo Fisher Scientific) according to the manufacturer’s protocol. The PCR reaction with a final volume of 10 *μ*l was performed at 95^∘^C for 10 min, 40 cycles of 95^∘^C for 15 sec and 60^∘^C for 1 min. The reactions were performed in triplicate using a 7900HT Fast Real-Time PCR System (Applied Biosystems, Thermo Fisher Scientific). The analyses were performed using R statistical computing environment according to the 2- *Δ*Ct method [25, 36]. The small non-coding nucleolar RNA RNU48 provided in the TaqMan® Control MicroRNA Assay kit (Thermo Fisher Scientific.) was used as housekeeping for the analysis.

### Cell proliferation, viability, cytotoxicity and apoptosis assays

Cell proliferation assays were carried out by xCELLigence RTCA DP Instrument (Roche Applied Science, Rotkreuz, Switzerland), using the corresponding E-Plate (Roche Applied Science). The experiments were performed following the manufacturer’s protocol. The xCELLigence system transforms automatically the impedance of electron flow caused by cells in a cell index (CI) value according to the formula CI = (impedance at time point n-impedance in the absence of cells) / nominal impedance value [37]. For the proliferation assay, concentrations of 8x10^3^ for all BC transfected cells were used and the CI value was monitored for 5 days, as previously reported [38]. The ApoTox-Glo Triplex Assay (Promega) was used to determine the cell viability, cytotoxicity, and apoptosis in transfected cells. Cells were seeded into 96-well culture plates and transfected as described above. After treatment, cells were incubated with 100 *μ*L fresh cell culture medium. At 24 h after transfection start, 20 *μ*L viability/cytotoxicity reagent was added to the cells. Following incubation for 1h, fluorescence was measured with the microplate multimode reader Varioskan™ (Thermo Fisher Scientific) at 495 nm for quantification of cell viability and at 535 nm for determination of cytotoxicity. Afterward, 100 *μ*L Caspase-Glo 3/7 Reagent was added to each well. Following incubation for 30 min, luminescence was further measured. The apoptosis process was also assayed by flow cytometry as previously described [39]. Cells transfected with miR-193, miR-210 inhibitors, or negative controls were plated onto a six-well plate at a density of 1x10^6^ cells/ well, allowed to adhere for at least 24 h and serum starved for 12 h. Cells were resuspended in the appropriate binding buffer, stained with FITC-conjugated Annexin V (BD Biosciences, San Jose, CA, USA) and propidium iodide (PI) at room temperature for 15 min and subsequently analyzed by flow cytometry in a BD FACSCanto II (BD Biosciences). Percentage of early (annexin V-FITC positive, PI negative) and late (annexin V-FITC positive, PI positive) apoptotic cells were determined by quadrant analysis of annexin V-FITC/PI plots using the software BD FACSDiva (BD Biosciences) following the manufacturer’s recommended protocol. A total of 2x10^4^ cells were evaluated in flow cytometry assays.

### Transwell migration and invasion assays

The transwell migration assay were performed using transwell chambers (8- *μ*m pore size; Corning, USA). Briefly, 8x10^5^ cells were plated in serum-free medium onto the upper compartment of the chamber. Medium containing 10% FBS was added to the lower compartment as a chemo-attractant, followed by an incubation of 24 h at 37^∘^C. Then, the porous inserts were removed and the cells that had migrated were fixed, stained and counted (magnification of x200). The experiments were all repeated at least three times. Cell imaging was analyzed by ImageJ software [40]. Cell invasion assay in negative controls and transfected cells was performed using a 24-well BD Biocoat Matrigel Invasion Chambers (BD Biosciences), with the same conditions as described for the migration assays.

### Statistical analysis

The results of all in vitro experiments, provided as continuous data, are expressed as the means ± standard deviations (SD) from three independent experiments. Statistical comparisons were calculated using Student’s t-test or one-way ANOVA (Analysis of variance) with post-hoc Tukey test for multiple comparisons and *P* ≤0.05 was considered. All the analyses were carried out using the R environment (version 3.2.3) [25].

## Results

### Identification of differentially expressed microRNAs in breast cancer cell lines and tissues

**Fig. 1:**
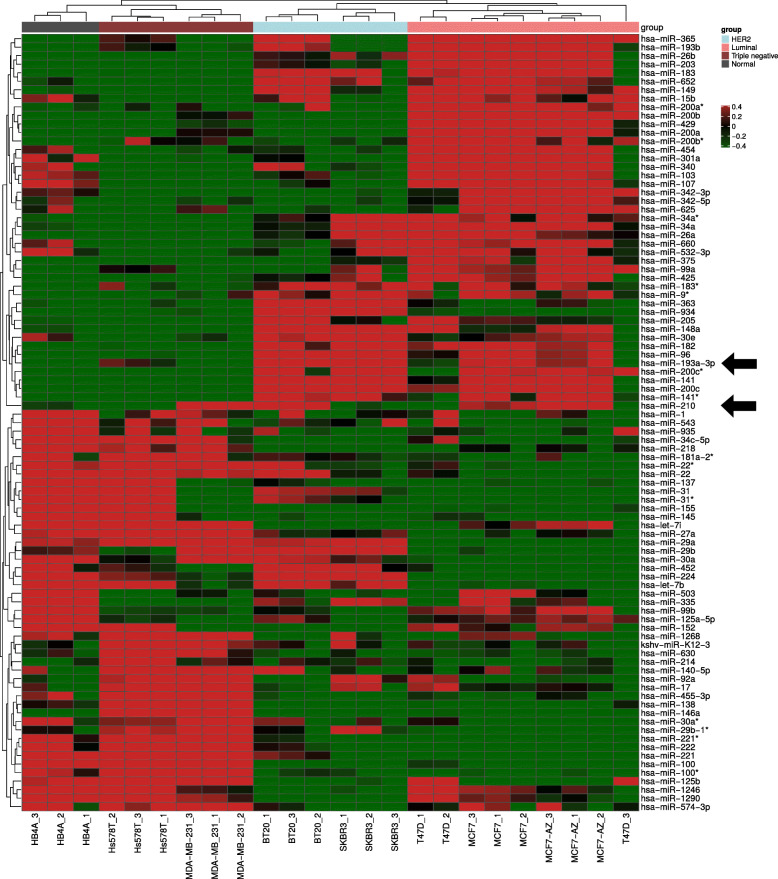
Hierarchical clustering analysis of breast cancer cell lines and normal HB4A cells. Unsupervised hierarchical cluster analysis representing the 92 miRNAs expressed in breast cancer cell lines versus normal HB4A cells

**Fig. 2:**
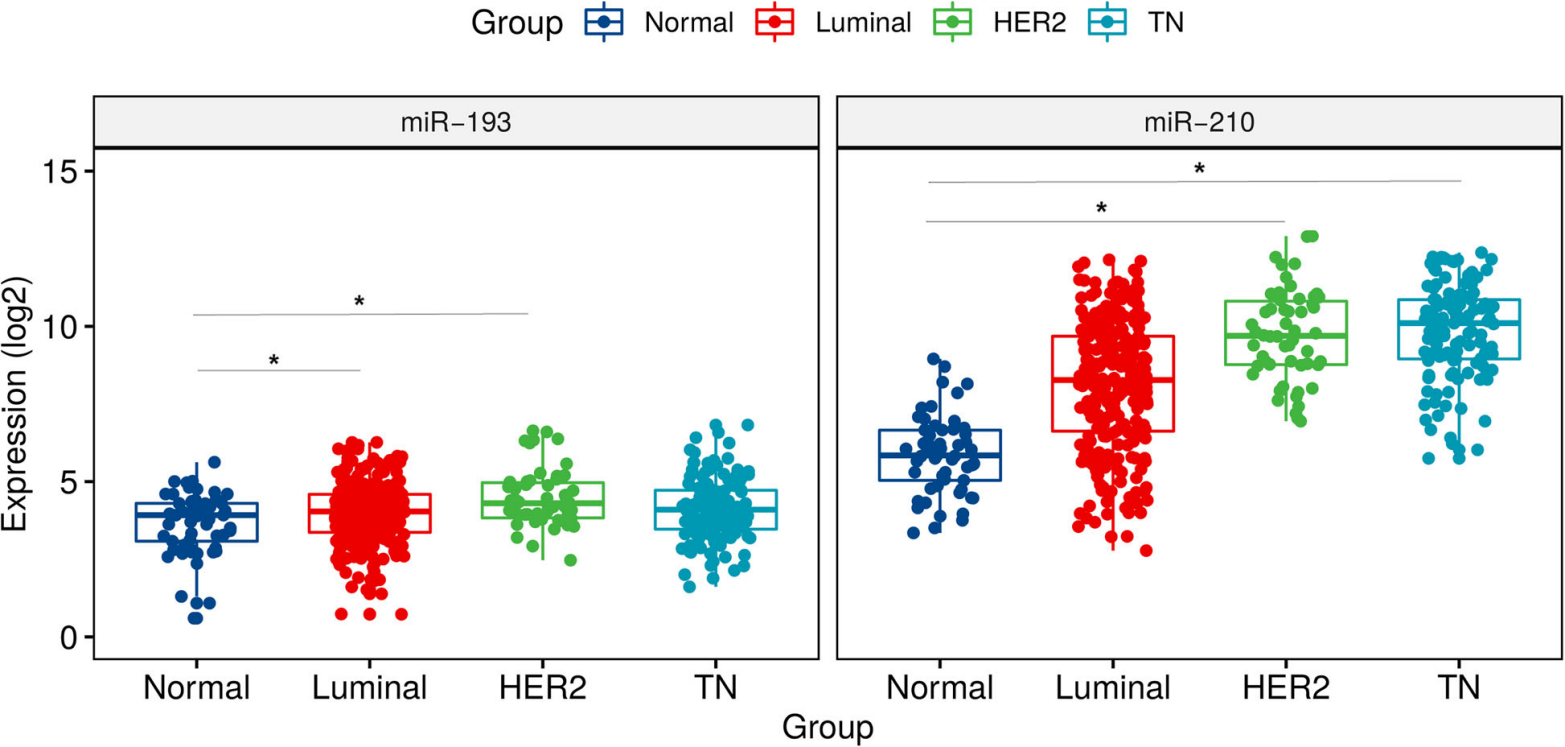
Validation of the selected microRNAs (hsa-miR-193a-3p and hsa-miR-210) expression in TCGA data. The log of the normalized expression values of miRNA data of TCGA from patients of different molecular subtypes were compared to expression levels from normal breast tissue samples. Mean ± SD are shown; *P ≤0.01

We identified a global miRNA profile using a panel of breast cancer cell lines from luminal (MCF-7, MCF-7/AZ and T47D), HER2 overexpressed (BT-20 and SKBR3), triple negative subtypes (MDA-MB-231 and Hs578T) and normal epithelial breast cancer cell line (HB4A). The flowchart of the study design is shown in Additional file 1: Figure S1A. Different transcripts derived from the same miRNAs based on our microarrays analysis were grouped together using the median. Ninety-one unique miRNAs were considered to be differentially expressed following several filtering steps in our analyses (Fig. 1). These criteria included a stringent multi-test analysis (ANOVA p ≤0.01 with Bonferroni correction), followed by filtering using a non-parametric test (rank products p ≤0.05 and pfp ≤0.05) selecting miRNAs with a fold change ≥2.0 in comparison to the normal HB4A cell line. Analysis of these 91 differentially expressed miRNAs showed that 32 were upregulated in comparison with the control cell line (Table 1). Biological information regarding these 32 miRNAs are displayed in Table 1. Since the miRbase version of microarrays is 12.0, the nomenclature from most updated version until now (v. 22.0), together with microRNA validation information, are also provided (Table 1). We then performed further functional assays on two miRNAs that were selected because they were upregulated in more than one BC molecular subtype (Additional file 1: Figure S1B). The miR-210 was upregulated in both the triple negative and in the HER2+ subtypes, while miR-193 was differentially expressed in the HER2+ and luminal subtypes. One representative cell lines of each subtype, MDA-MB-231, BT-20 and MCF-7, respectively, were selected for further evaluations (Additional file 1: Figure S1B). Analysis of expression levels in the TCGA BC datasets confirmed that miR-210 and miR-193 were the highest ranking miRNAs, with expression levels that were significantly correlated with their respective associated molecular subtypes (triple-negative and HER2+ for miR-210 and miR-193 for luminal and HER2+). The expression level of miR-210 was significantly increased in BC tumors in comparison to the normal controls (Fig. 2). Similarly, the expression of miR-193 was also upregulated in BC when compared to the normal epithelial cells, HB4A (Fig. 2). Collectively, our results indicate that miR-210 is upregulated in BC and let us hypothesize that downregulation by silencing assays of this miRNA could have an effect on the cells of different molecular subtypes.

**Table 1:** Deregulated miRNA in breast cancer cell lines compared to normal HB4A cells

Microarray ID	Probe sequence	MIMAT ID	miRbase (rel. 22)	Validation information [*]
hsa-miR-141	CCATCTTTACCAGACA	MIMAT0000432	hsa-miR-141-3p	experimental; cloned
hsa-miR-141*	TCCAACACTGTACTGGAA	MIMAT0004598	hsa-miR-141-5p	experimental; cloned
hsa-miR-148a	ACAAAGTTCTGTAGTGCACT	MIMAT0000243	hsa-miR-148a-3p	experimental; cloned, Northern
hsa-miR-149	GGGAGTGAAGACACGGAG	MIMAT0000450	hsa-miR-149-5p	experimental; cloned
hsa-miR-182	AGTGTGAGTTCTACCAT	MIMAT0000259	hsa-miR-182-5p	experimental; cloned
hsa-miR-183	AGTGAATTCTACCAGTGCCA	MIMAT0000261	hsa-miR-183-5p	experimental; cloned
hsa-miR-183*	TTATGGCCCTTCGGT	MIMAT0004560	hsa-miR-183-3p	experimental; cloned
hsa-miR-193a-3p	ACTGGGACTTTGTAGGC	MIMAT0000459	hsa-miR-193a-3p	experimental; cloned
hsa-miR-193b	AGCGGGACTTTGAGGG	MIMAT0002819	hsa-miR-193b-3p	experimental; array-cloned, cloned
hsa-miR-200a	ACATCGTTACCAGACAGT	MIMAT0000682	hsa-miR-200a-3p	experimental; cloned
hsa-miR-200a*	TCCAGCACTGTCCGGT	MIMAT0001620	hsa-miR-200a-5p	experimental; cloned
hsa-miR-200b	TCATCATTACCAGGCAG	MIMAT0000318	hsa-miR-200b-3p	experimental; Northern, cloned
hsa-miR-200b*	TCCAATGCTGCCCAG	MIMAT0004571	hsa-miR-200b-5p	experimental; cloned
hsa-miR-200c	TCCATCATTACCCGG	MIMAT0000617	hsa-miR-200c-3p	experimental; cloned, Northern
hsa-miR-200c*	CCAAACACTGCTGGGTA	MIMAT0004657	hsa-miR-200c-5p	experimental; cloned
hsa-miR-203	CTAGTGGTCCTAAACATT	MIMAT0000264	hsa-miR-203a-3p	experimental; cloned
hsa-miR-205	CAGACTCCGGTGGAAT	MIMAT0000266	hsa-miR-205-5p	experimental; cloned
hsa-miR-210	TCAGCCGCTGTCACAC	MIMAT0000267	hsa-miR-210-3p	experimental; cloned, Illumina
hsa-miR-26a	AGCCTATCCTGGATT	MIMAT0000082	hsa-miR-26a-5p	experimental; cloned, Northern
hsa-miR-26b	ACCTATCCTGAATTACTTGA	MIMAT0000083	hsa-miR-26b-5p	experimental; cloned, Northern
hsa-miR-34a	ACAACCAGCTAAGACACTGC	MIMAT0000255	hsa-miR-34a-5p	experimental; cloned
hsa-miR-34a*	AGGGCAGTATACTTGCTG	MIMAT0004557	hsa-miR-34a-3p	experimental; cloned
hsa-miR-363	TACAGATGGATACCGTGCA	MIMAT0000707	hsa-miR-363-3p	experimental; array-cloned, cloned
hsa-miR-365	ATAAGGATTTTTAGGGGCATTA	MIMAT0000710	hsa-miR-365a-3p	experimental; cloned, array-cloned
hsa-miR-375	TCACGCGAGCCGAAC	MIMAT0000728	hsa-miR-375-3p	experimental; cloned
hsa-miR-425	TCAACGGGAGTGATCGTG	MIMAT0003393	hsa-miR-425-5p	experimental; cloned
hsa-miR-429	ACGGTTTTACCAGACAGTA	MIMAT0001536	hsa-miR-429	experimental; cloned
hsa-miR-652	CACAACCCTAGTGGC	MIMAT0003322	hsa-miR-652-3p	experimental; Microarray, SAGE, cloned
hsa-miR-9*	ACTTTCGGTTATCTAGCTT	MIMAT0000442	hsa-miR-9-3p	experimental; cloned
hsa-miR-934	CCAGTGTCTCCAG	MIMAT0004977	hsa-miR-934	experimental; cloned
hsa-miR-96	AGCAAAAATGTGCTAGTGCCA	MIMAT0000095	hsa-miR-96-5p	experimental; cloned
hsa-miR-99a	CACAAGATCGGATCTACGG	MIMAT0000097	hsa-miR-99a-5p	experimental; cloned

According to miRbase

### Target prediction and enrichment analysis

We used a mRNA microarray to screen for aberrant expression of mRNAs in the same BC cells used for our initial miRNA assays. The candidate genes were first screened using several miRNA target prediction engines provided by mirDip data repository, and then the top differentially expressed target mRNAs for miR-210 and miR-193 were selected. KEGG pathway enrichment analysis was obtained for the predicted mRNAs (Table 2). This analysis showed that the following pathways: Direct p53 effectors, signaling by interleukins, PI3K-Akt signaling, generic transcription pathway,FoxO signaling pathway, hippo signaling pathway and regulation of nuclear SMAD2/3 signaling could all be associated with these target miRNAs, after filtering for breast neoplasia, according to ReactomeFI analysis. The mRNA targets identified in the selected pathways were evaluated according to their differential expression in the TCGA data and the genes that showed the same profile were selected. Between them, the genes *TNFRSF10C/D* and *CCND1* were the top ranked and are highlighted in bold in Table 2.

**Table 2:** Top pathways of miRNA targets predicted by mirDIP analysis

Pathway	FDR-corrected *P*-value	Genes
Direct p53 effectors	2,54E-14	*TRRAP,SERPINE1,CREBBP,CASP10,SERPINB5,HTT,IGFBP3,HIC1,MCL1*
		***TNFRSF10C***,TNFRSF10B,***TNFRSF10D***,*BCL6,SP1,MDM2,BCL2L1,PTEN*
		*APAF1,MET,TP53INP1,E2F1,BAK1,PML,APC,CCNK,EPHA2,LIF,SMARCA4*
		*TP73,PRMT1,FOXA1,EP300,HGF,TSC2,NDRG1,CAV1,VCAN*
Signaling by Interleukins	2,54E-14	*FRS2,GATA3,HIF1A,PSMF1,IL22RA1,COL1A2,JAK2,DUSP6,DUSP7,NRAS*
		*ALOX5,STAT6,STAT1,STAT3,DAB2IP,CSF1,IRS2,RORA,FGF1,SQSTM1*
		*HSP90B1,SOCS3,SOCS1,MCL1,KL,BCL6,NF1,BCL2L1,FLT3,KSR1,NRG1*
		*TNFRSF1B,PIK3CA,MET,CRK,NOD2,ANGPT1,CRKL,SOX2,BTRC,AKAP9*
		*STAT5B,LIF,PTK2,SMARCA4,GRB2,SYK,OSMR,IL1A,KIT,RHOU,MAP3K3*
		*OSM*,***CCND1***,*HGF,LIFR,POU2F1,IL7,FGFR1,CXCL2,MMP9,YWHAZ,CBL*
		*ERBB4,PTK2B,MAPK1,NCAM1,CAMK2G,KRAS*
PI3K-Akt signaling pathway	5,83E-13	*AKT2,MYB,PRKCA,NGFR,COL1A2,JAK2,NRAS,CSF1,FGF1,HSP90B1,BCL2L11*
		*MCL1,CDK6,MDM2,BCL2L1,FLT1,ITGB3,PTEN,PIK3CG,VWF,ITGA2,PIK3CA*
		*ITGA6,MET,EIF4E,ANGPT1,PPP2R1B,EPHA2,LPAR1,LPAR3,PTK2,GRB2*
		*BRCA1,SYK,OSMR,KIT,SGK1,RXRA,OSM,IGF1R,CCND2*,***CCND1***,*HGF,TSC2*
		*TSC1,IL7,FGFR1,PDPK1,YWHAZ,RBL2,LAMA4,THBS2,THBS1,MAPK1,KRAS*
Generic Transcription Pathway	5,83E-13	*SERPINE1,GLS,MED1,BARD1,CREBBP,NR2F1,CASP10,TEAD1,NCOR2,NCOR1*
		*RARA,PPARA,RICTOR,RORA,CTGF,YY1,IGFBP3,NOTCH2,NOTCH1*,***TNFRSF10C***
		*TNFRSF10B,ESRRG*,***TNFRSF10D***,*BCL6,SP1,MDM2,PIN1,PTEN,APAF1,GLS2*
		*TP53INP1,E2F1,E2F4,PML,CCNK,CCNC,CHEK1,RUNX2,ATM,TP73,BRCA1*
		*NCOA2,PRMT1,KIT,SGK1,RXRA,NR3C1,EP300,TSC2,TSC1,FANCD2,NDRG1*
		*SMAD2,TFAP2A,TFAP2C,SMAD4,SMAD3,ESR1,NR4A3,THRB,PDPK1,YWHAZ*
		*RBL2,RBL1,NR2C1,MTA2,MAPK14,MAPK11*
FoxO signaling pathway	5,83E-13	*AKT2,CREBBP,PLK1,NRAS,STAT3,IRS2,BCL2L11,BCL6,MDM2,PTEN,PIK3CG*
		*PIK3CA,CSNK1E,GRB2,ATM,PRMT1,SGK1,IGF1R,CCND2*,***CCND1***,*EP300*
		*SMAD2,SMAD4,TGFB2,SMAD3,PDPK1,TGFBR1,RBL2,MAPK8,MAPK1*
		*MAPK14,MAPK10,MAPK11,KRAS*
Hippo signaling pathway	6,85E-11	*SERPINE1,PRKCZ,FZD1,TEAD1,WNT7A,FGF1,CTGF,DVL1,NF2,BMPR2,APC*
		*SOX2,RASSF1,PPP2R1B,BTRC,CSNK1E,TP73,CTNNB1,WWC1,CCND2*,***CCND1***
		*WNT5A,SMAD2,SMAD4,TGFB2,SMAD3,BMP6,DLG1,AXIN2,YWHAZ,TGFBR1*
		*LATS1,TCF7L2*
Regulation of nuclear	2,51E-06	*SERPINE1,GATA3,CREBBP,COL1A2,NCOR1,CBFB,SP1*,***RUNX3***,*RUNX2,RUNX1*
SMAD2/3 signalin		*NCOA2,NR3C1,EP300,SMAD2,SMAD4,SMAD3,ESR1*

In bold are the genes with validation according to differential expression in the TCGA data

### Functional in vitro assays

As a first approach to investigate the functional significance of miRNAs that are downregulated in BC, cell lines representative of specific BC subtypes were transiently transfected with inhibitors of miR-210, miR-193, or negative control. Transfection efficiencies of miR-210 and miR-193 in BC cells was confirmed by RT-qPCR and only the assays encompassing ≥70% of inhibition were further evaluated. To determine whether the reduction of expression of both miRNAs could modify BC cells behavior, cells of different subtypes were transfected with specific inhibitors and then effects on viability, cytotoxicity and apoptosis were measured. No changes were observed in cell viability for either miRNA in comparison to cells transfected with the negative controls (Additional file 2: Figure S2). Conversely, inhibition of both miRNAs did not decrease the percentage of cytotoxicity in the times analyzed (Additional file 3: Figure S3). The analysis of apoptosis did not show any changes either (Additional file 1: Figure S4). Since some cell lines, such as MCF-7 do not express caspase 3 [41], additional experiments were conducted to evaluate apoptosis by the Annexin V and propidium iodide staining methodology. No significant differences were found for levels of apoptosis after miR-210 and miR-193 transfections in comparison to controls (Additional file 5: Figure S5) and this led us to rule out the occurrence of viability, cytotoxicity and especially apoptosis. Through the *TNFRSF10C* and *TNFRSF10D* mRNA expression was inversely correlated with expression levels of miR-193 and miR-210 in breast cell lines and breast cancer patients, respectively. Since these targets mRNAs are known inhibitors of apoptosis, these results suggest that they could potentially inhibit the apoptosis initiation mechanisms after selected miRNA silencing.

### Alterations in proliferation, invasion and migration

**Fig. 3:**
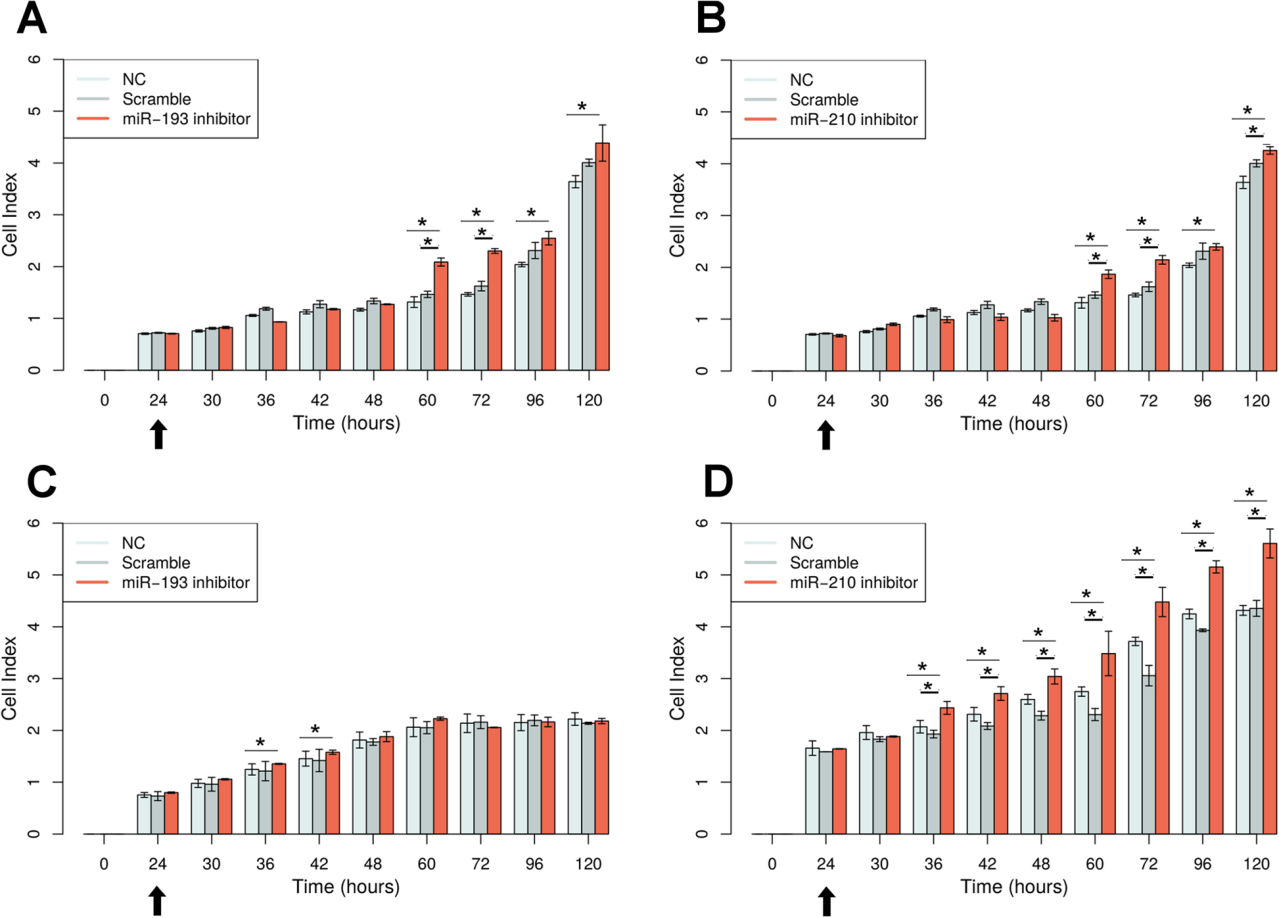
Real-time proliferation analysis using xCELLigence system. The miRNA inhibition time is indicated by arrows. **a** Silencing of miR-193 in BT-20 cells; **b** Silencing of miR-210 in BT-20 cells; **c** Silencing of miR-193 in MCF-7 cells; **d** Silencing of miR-210 in MDA-MB-231 cells

Our results revealed alterations in cell proliferation and in different time points for both miRNAs in all cells evaluated. The most evident effects were associated with cell proliferation after miR-210 silencing in triple negative subtype cell line MDA-MB-231. In silico analyses (see Table 2) predicted *RUNX3* as a target of miR-210. These results showed involvement of *RUNX3* whose expression was inversely correlated for this miRNA. Downregulation of miR-210 expression in MDA-MB-231 cells significantly increased cell proliferation, as demonstrated by xCELLigence assays (Fig. 3). These data suggest that miR-210 could affect cell proliferation in triple-negative BC cells. No effect was observed for migration and invasion analysis in all BC cells of different molecular subtypes studied (Additional file 6: Figure S6 and Additional file 7: Figure S7). Taken together, these data show that both miRNAs presented effect in proliferation of all cells at different times and miR-210 presented the higher effect of the proliferation on triple negative cells in vitro.

## Discussion

Breast cancer (BC) is a heterogeneous malignancy with complex biology that influences the choices of targeted therapies [3]. This disease is presently characterized as comprising five main intrinsic molecular subtypes [9]. However, in spite of the extensive characterization of BC, novel biomarkers are still needed to provide more comprehensive molecular classification of BC for improved precision medicine.

There are an abundance of reports elucidating the mechanisms and roles of miRNAs as novel and stable biomarkers involved in a broad range of tumors, including BC [42, 43]. In this work, our experimental design allowed us to select for miRNAs that may play a regulatory role within the established molecular subtypes of BC. We performed a screening in BC cell lines and TCGA patients that demonstrated the involvement of miR-193 and miR-210 in different molecular subtypes of BC. Moreover, we observed that miR-210 plays a regulatory role in BC proliferation after its inhibition, especially in the most aggressive triple negative MDA-MB-231 cells.

Previous studies have identified miR-210 with high expression in a variety of tumor cells under hypoxic conditions [44, 45]. This miRNA also exhibits oncogenic properties and its upregulation has been recently identified on multiple human cancers, such as colorectal, bone metastatic prostate, ovarian and lung cancer, among others [46–49]. The miR-210 experimentally validated targets have provided new insights about miR-210 functional roles, including regulation of mitochondrial metabolism, cell cycle control, angiogenesis, apoptosis, and DNA damage repair [44, 50]. It has also been identified as a serum marker in several types of cancer, which suggests that miR-210 could be a biomarker for early detection in metastatic tumors [51]. In BC, miR-210 expression seems to be correlated with *VEGF* expression, indicating a possible role in tumor angiogenesis [52]. A recent meta-analysis has described miR-210 as upregulated in most studies [53]. With respect to the expression of miR-210 in BC cell lines, Shi et al. [54] compared the transcriptome of MCF-7 and MDA-MB-231 cells using next generation sequencing, showing that the expression of miR-210 in the triple negative MDA-MB-231 cell line can be four times higher than luminal MCF-7. In concordance with this finding, we used MDA-MB-231 and BT-20 in our functional assays because of higher expression of miR-210 in these cells. This miRNA was also upregulated in triple-negative breast cancer patients and was also correlated with a poor prognosis and metastasis [55–57].

Among the mRNA targets of miR-210 that we identified, the *RUNX3* gene was recently identified as a direct target in endothelial cells, affecting proliferation, migration and invasion processes on them [58]. In BC, this gene was initially described as a tumor suppressor and related to estrogen receptor signaling [59, 60]. However, there is also evidence of its effect on triple-negative breast cancer cell proliferation, as induced by miRNAs [61]. These obsevations support the findings that alterations on the expression of this target, as potentially correlated with miR-210, could be candidate regulatory miRNA affecting BC cell proliferation, with implications especially for the most aggressive triple negative subtype.

The other regulatory miRNA candidate we identified by a combination of global expression analysis and functional assays was miR-193a-3p. This miRNA has been also described in a broad range of tumors, such as lung, colorectal, gastric, ovarian cancer, among others [62]. Among validated targets for this microRNA, the *PTEN* gene was associated in gastric cancer and renal cell carcinoma [63, 64] and *ERBB4* in lung cancer [65], suggesting that regulation by miR-193 could act as a tumor suppressor. Other specific functional targets associated with miR-193a-3p include pathways that impact cell proliferation, invasion, migration and metastasis [62]. In breast cancer, a member of miR-193 family, the miR-193b, directly targets estrogen receptor (ER) suppressing the cancer cell growth [66]. This observation agrees with our findings regarding increase of proliferation in MCF-7 cells of luminal subtype. Furthermore, a significantly decreased expression in miR-193b was observed in triple negative BC cell lines in comparison to non-triple negative and normal cells [67], in concordance with our findings that there was higher expression of miR-193 in luminal and HER-2+ BC cells. Between the top mRNAs with anti-correlated expression to miR-193 we identified, the *CCND1* to be associated with cell proliferation as a direct target in melanoma, prostate, ovarian and even in breast cancer [68–71].

Our results also demonstrated there was no apoptotic activity after the silencing of both miRNAs. However, we found strong evidence that the overexpression of TRAIL decoy receptors *TNFRSF10D* and *TNFRSF10C* could be related to miR-210 and miR-193 downregulation, respectively. TRAIL molecules are members of TNF family and can induce apoptosis selectively in cancer cells, and they are considered as promising anticancer agents [72, 73]. The TRAIL apoptotic process occurs by its binding to death receptors but the competitive interaction with decoy receptors 1 (*DcR1*/*TRAILR3*/*TNFRSF10C*) and 2 (*DcR2*/*TRAILR4*/*TNFRSF10D*) can induce an inhibitory effect [72]. The overexpression of *TNFRSF10D* was shown to be able to protect cells against apoptosis and its expression was associated with BC risk [74, 75]. Furthermore, aberrant promoter methylation of TRAIL decoy receptors can be affected by *DNMT3A* which can be a direct target of microRNAs [76, 77].

Thus, in this study we identified a molecular signature of miRNAs in BC cell lines and explored the expression and functional role of two promising regulatory biomarkers: miR-210 and miR-193 that could affect mRNA expression in different molecular subtypes.

## Conclusion

Collectively our findings show that two miRNAs (miR-210 and miR-193) as associated with specific BC molecular subtypes and may be mediating expression of genes involved in pathways of clinical relevance in BC. Further studies are necessary to validate their targets and clinical utility.

## Supplementary Information

**Additional file 1** Overview of the analysis of microarray data and functional assays of breast cancer cell lines. In (A) Pipeline used to identify deregulated miRNAs. In (B) Cell lines and microRNAs selected for further functional analysis.

**Additional file 2** Effect of microRNA inhibition on the viability of breast cancer cells. Cell viability was evaluated using the ApoTox-Glo Triplex Assay Kit as described in Materials and Methods. Mean ± SD of three independent experiments are shown; *P ≤0.01. RFU, relative fluorescence units. (A) Silencing of miR-193 in BT-20 cells; (B) Silencing of miR-210 in BT-20 cells; (C) Silencing of miR-193 in MCF-7 cells; (D) Silencing of miR-210 in MDA-MB-231 cells.

**Additional file 3** Effect of microRNA inhibition on the citotoxicity of breast cancer cells. Citotoxicity was evaluated using the ApoTox-Glo Triplex Assay Kit as described in Materials and Methods. Mean ± SD of three independent experiments are shown; *P ≤0.01. RFU, relative fluorescence units. (A) Silencing of miR-193 in BT-20 cells; (B) Silencing of miR-210 in BT-20 cells; (C) Silencing of miR-193 in MCF-7 cells; (D) Silencing of miR-210 in MDA-MB-231 cells.

**Additional file 4** Effect of microRNA inhibition on the apoptosis of breast cancer cells. Apoptosis was evaluated using the ApoTox-Glo Triplex Assay Kit as described in Materials and Methods. Mean ± SD of three independent experiments are shown; *P ≤0.01. RLU, relative luminescence units. (A) Silencing of miR-193 in BT-20 cells; (B) Silencing of miR-210 in BT-20 cells; (C) Silencing of miR-193 in MCF-7 cells; (D) Silencing of miR-210 in MDA-MB-231 cells.

**Additional file 5** Apoptosis analysis of mDA-MB-231 cells at 24 h post-transfection of negative control. In (A), scramble (B) or with miR-210 (C), and 72h post-transfection of negative control (D), scramble (E) or with miR-210 (F), as evaluated by Annexin V and propidium iodide staining and FACS analysis. The percentage of necrotic (Q1), late apoptotic (Q2), viable (Q3), and early apoptotic (Q4) cells are shown in the corresponding quadrants.

**Additional file 6** Effect of microRNA inhibition on the migration of breast cancer cells, as evaluated by transwell assay. Mean ± SD of three independent experiments are shown; *P ≤0.01. (A) Silencing of miR-193 in BT-20 cells; (B) Silencing of miR-210 in BT-20 cells; (C) Silencing of miR-193 in MCF-7 cells; (D) Silencing of miR-210 in MDA-MB-231 cells.

**Additional file 7** Effect of microRNA inhibition on the invasion of breast cancer cells, as evaluated by transwell assay. Mean ± SD of three independent experiments are shown; *P ≤0.01. (A) Silencing of miR-193 in BT-20 cells; (B) Silencing of miR-210 in BT-20 cells; (C) Silencing of miR-193 in MCF-7 cells; (D) Silencing of miR-210 in MDA-MB-231 cells.

## Data Availability

All data used and analyzed during this study are available from the corresponding author on reasonable request.

## Abbreviations

ANOVA: Analysis of Variance
BC: Breast cancer
CI: Cell Index
FBS: Fetal Bovine Serum
miRNA: microRNA
TCGA: The Cancer Genome Atlas
