# Supplementary figures and images for: Integrated analysis of mRNA and miRNA profiles revealed the role of miR-193 and miR-210 as potential regulatory biomarkers in different molecular subtypes of breast cancer
Source: BMC Cancer. 2021 Jan 18;21:76. doi: 10.1186/s12885-020-07731-2 (PMC7814437; doi:10.1186/s12885-020-07731-2)

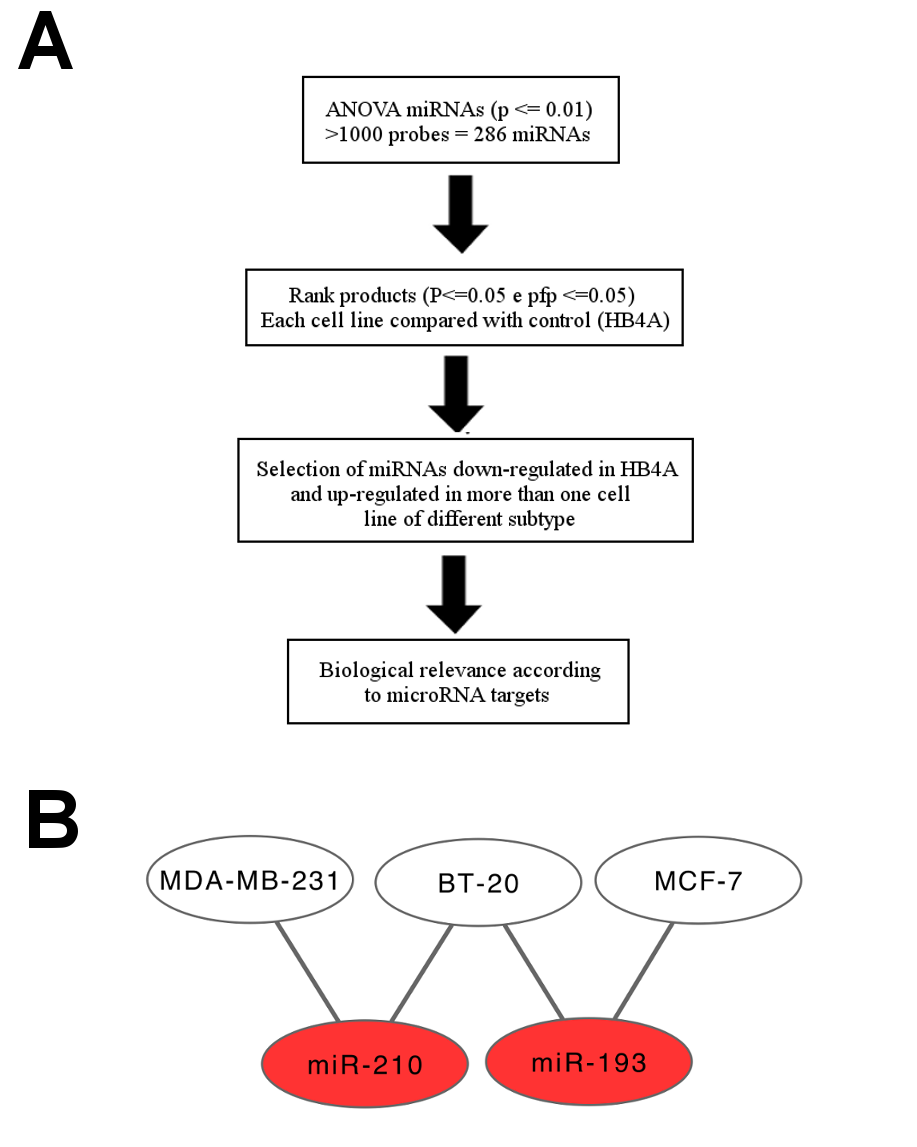

Supplement: Supplementary file 1 — Additional file 1 Overview of the analysis of microarray data and functional assays of breast cancer cell lines. In (A) Pipeline used to identify deregulated miRNAs. In (B) Cell lines and microRNAs selected for further functional analysis. [file 12885_2020_7731_MOESM1_ESM.tiff]

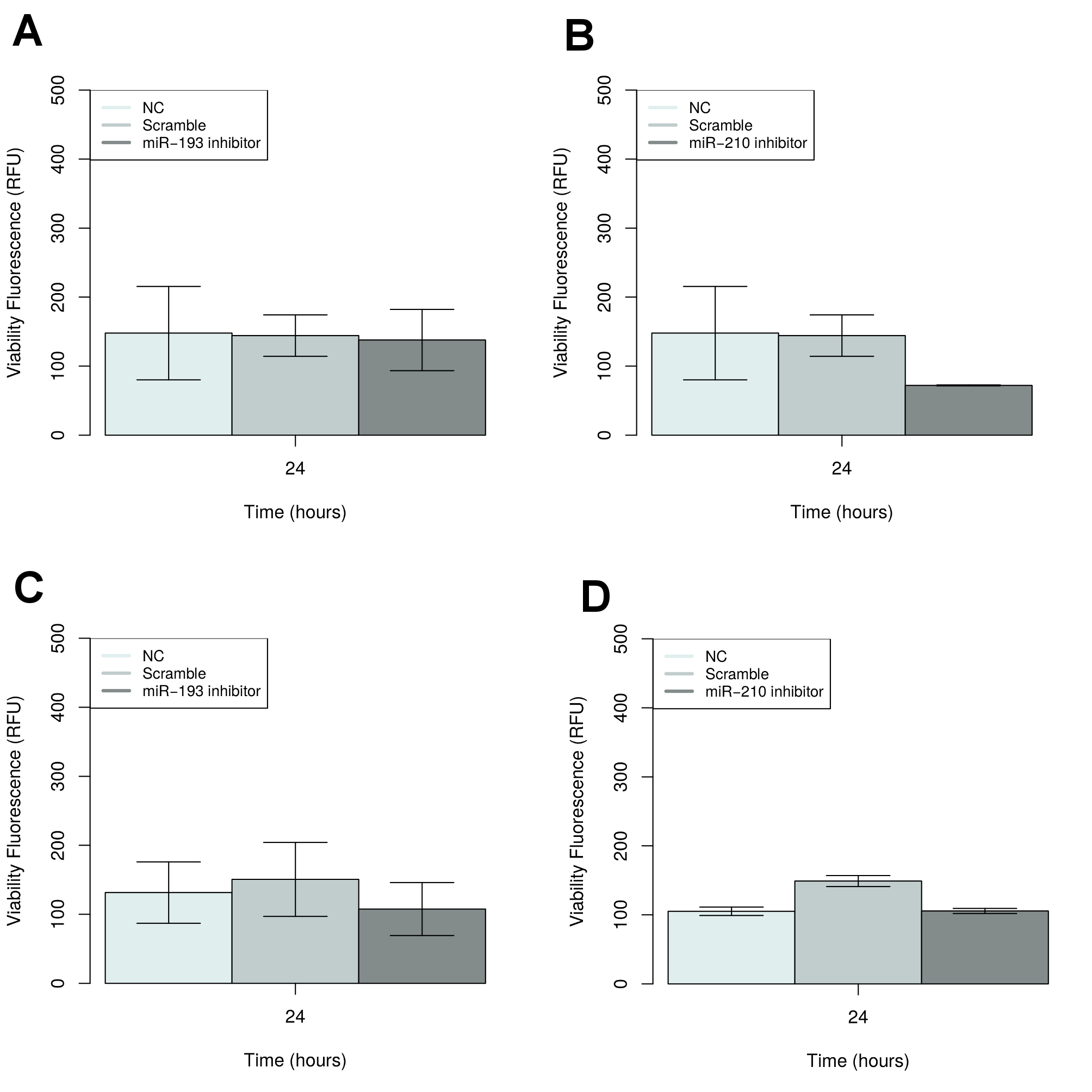

Supplement: Supplementary file 2 — Additional file 2 Effect of microRNA inhibition on the viability of breast cancer cells. Cell viability was evaluated using the ApoTox-Glo Triplex Assay Kit as described in Materials and Methods. Mean ± SD of three independent experiments are shown; *P ≤0.01. RFU, relative fluorescence units. (A) Silencing of miR-193 in BT-20 cells; (B) Silencing of miR-210 in BT-20 cells; (C) Silencing of miR-193 in MCF-7 cells; (D) Silencing of miR-210 in MDA-MB-231 cells. [file 12885_2020_7731_MOESM2_ESM.tiff]

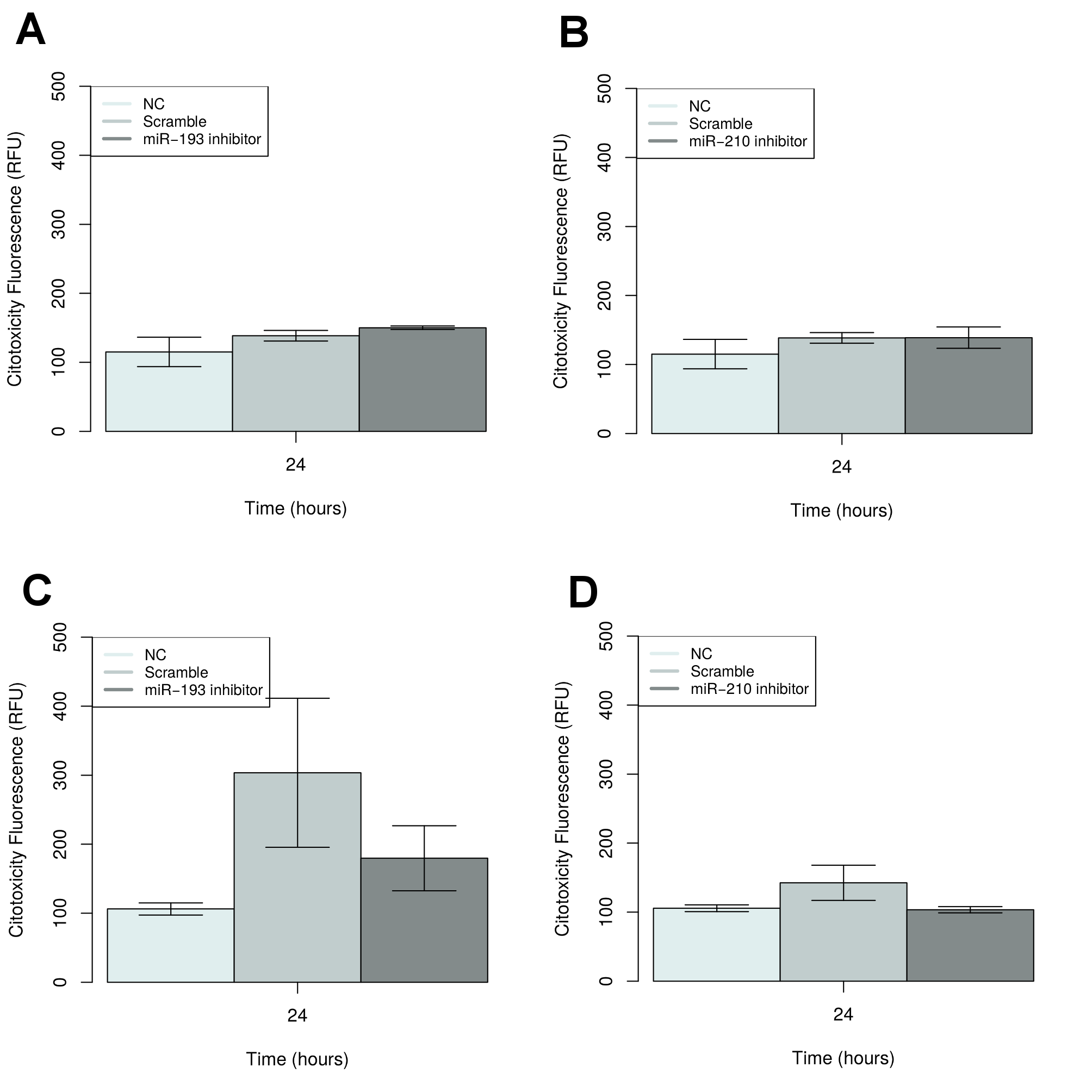

Supplement: Supplementary file 3 — Additional file 3 Effect of microRNA inhibition on the citotoxicity of breast cancer cells. Citotoxicity was evaluated using the ApoTox-Glo Triplex Assay Kit as described in Materials and Methods. Mean ± SD of three independent experiments are shown; *P ≤0.01. RFU, relative fluorescence units. (A) Silencing of miR-193 in BT-20 cells; (B) Silencing of miR-210 in BT-20 cells; (C) Silencing of miR-193 in MCF-7 cells; (D) Silencing of miR-210 in MDA-MB-231 cells. [file 12885_2020_7731_MOESM3_ESM.tiff]

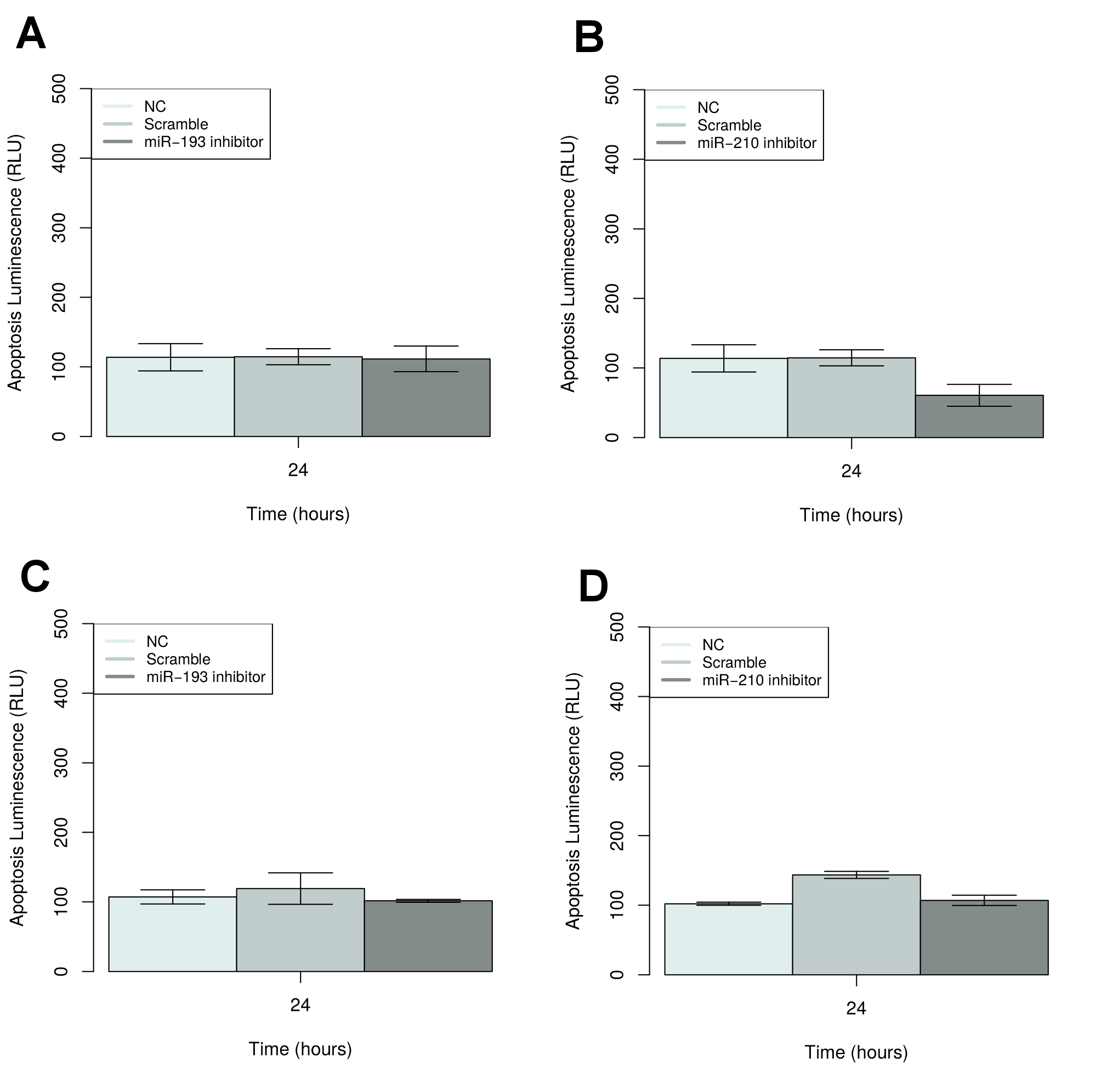

Supplement: Supplementary file 4 — Additional file 4 Effect of microRNA inhibition on the apoptosis of breast cancer cells. Apoptosis was evaluated using the ApoTox-Glo Triplex Assay Kit as described in Materials and Methods. Mean ± SD of three independent experiments are shown; *P ≤0.01. RLU, relative luminescence units. (A) Silencing of miR-193 in BT-20 cells; (B) Silencing of miR-210 in BT-20 cells; (C) Silencing of miR-193 in MCF-7 cells; (D) Silencing of miR-210 in MDA-MB-231 cells. [file 12885_2020_7731_MOESM4_ESM.tiff]

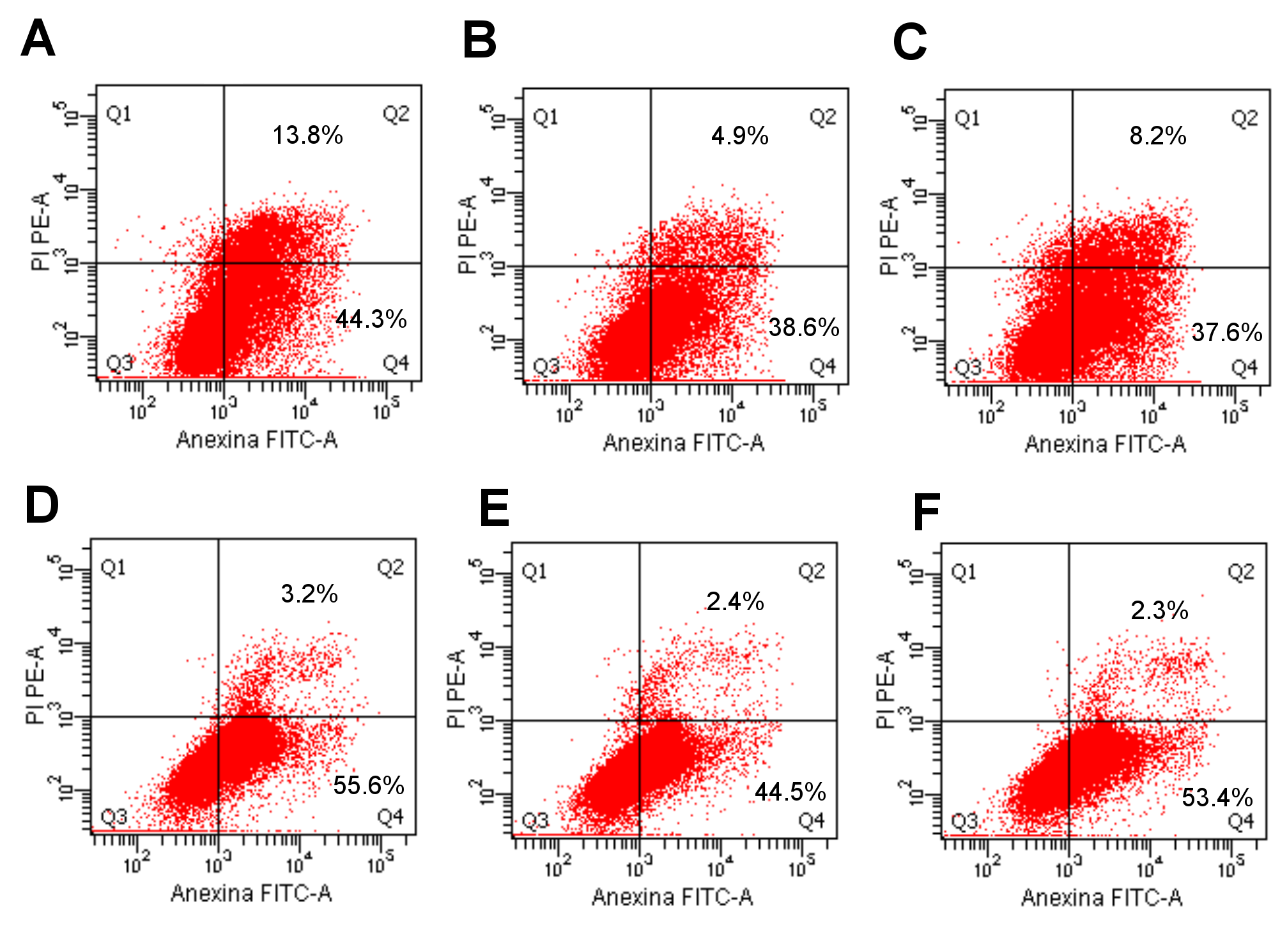

Supplement: Supplementary file 5 — Additional file 5 Apoptosis analysis of mDA-MB-231 cells at 24 h post-transfection of negative control. In (A), scramble (B) or with miR-210 (C), and 72h post-transfection of negative control (D), scramble (E) or with miR-210 (F), as evaluated by Annexin V and propidium iodide staining and FACS analysis. The percentage of necrotic (Q1), late apoptotic (Q2), viable (Q3), and early apoptotic (Q4) cells are shown in the corresponding quadrants. [file 12885_2020_7731_MOESM5_ESM.tiff]

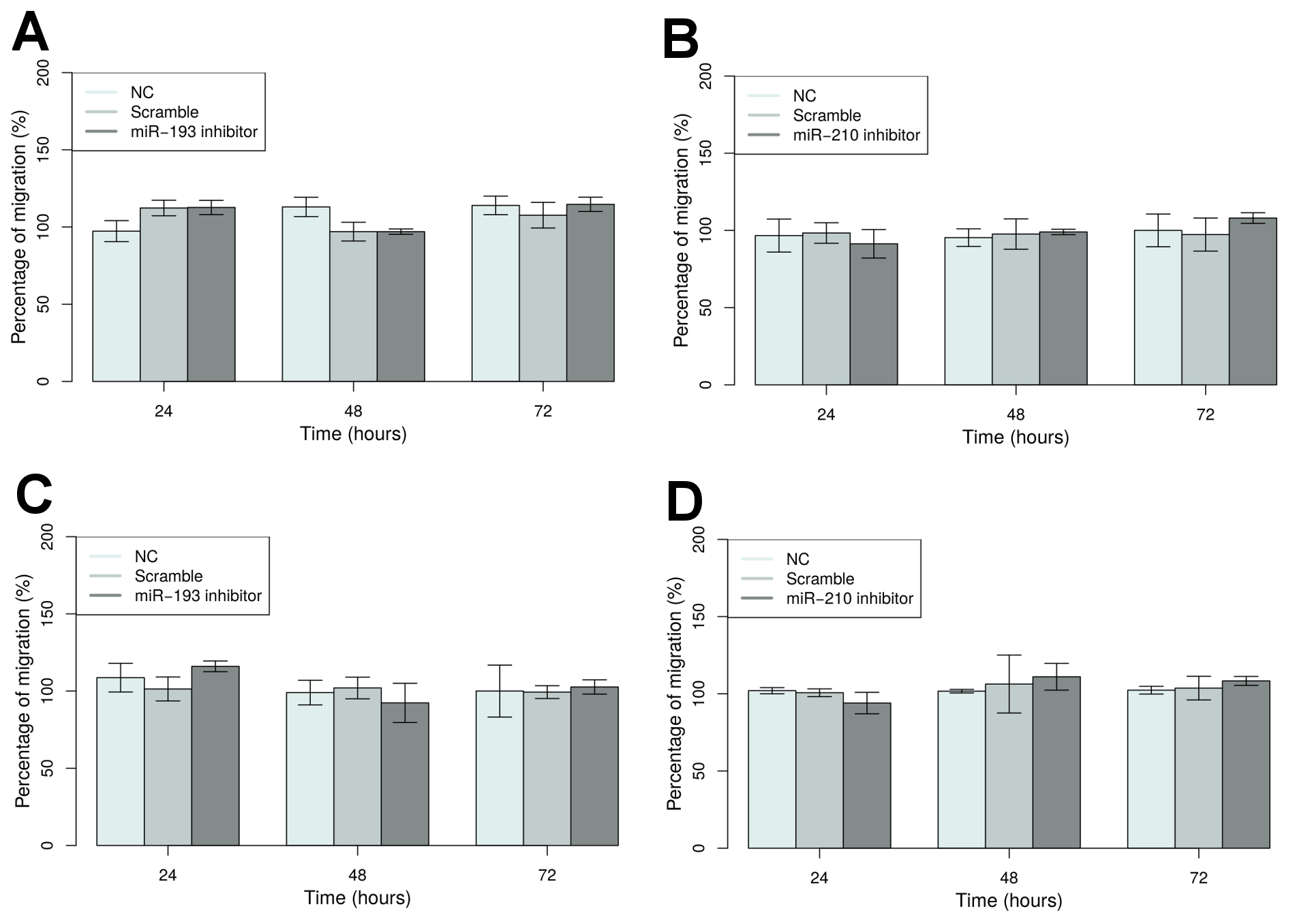

Supplement: Supplementary file 6 — Additional file 6 Effect of microRNA inhibition on the migration of breast cancer cells, as evaluated by transwell assay. Mean ± SD of three independent experiments are shown; *P ≤0.01. (A) Silencing of miR-193 in BT-20 cells; (B) Silencing of miR-210 in BT-20 cells; (C) Silencing of miR-193 in MCF-7 cells; (D) Silencing of miR-210 in MDA-MB-231 cells. [file 12885_2020_7731_MOESM6_ESM.tiff]

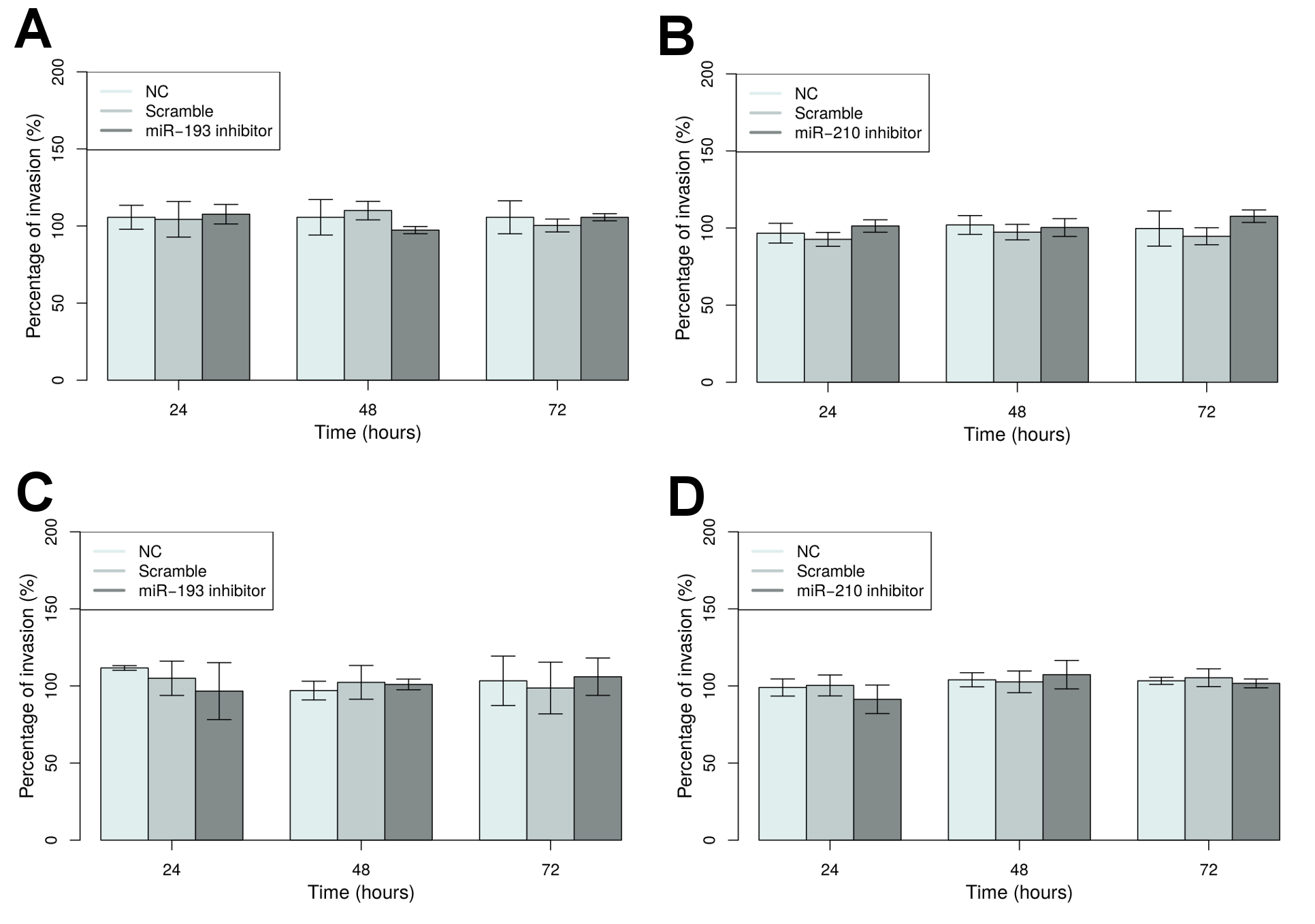

Supplement: Supplementary file 7 — Additional file 7 Effect of microRNA inhibition on the invasion of breast cancer cells, as evaluated by transwell assay. Mean ± SD of three independent experiments are shown; *P ≤0.01. (A) Silencing of miR-193 in BT-20 cells; (B) Silencing of miR-210 in BT-20 cells; (C) Silencing of miR-193 in MCF-7 cells; (D) Silencing of miR-210 in MDA-MB-231 cells. [file 12885_2020_7731_MOESM7_ESM.tiff]
